# Involvement of pre- and postsynaptic NMDA receptors at local circuit interneuron connections in rat neocortex

**DOI:** 10.1016/j.neuroscience.2012.10.012

**Published:** 2013-01-03

**Authors:** C.L. De-May, A.B. Ali

**Affiliations:** The School of Pharmacy, University College London, 29-39 Brunswick Square, London WC1N 1AX, UK

**Keywords:** AHP, after-hyperpolarisation, CV^−2^, inverse square of the coefficient of variation, d-AP5, d-(−)-2-amino-5-phosphonopentanoic acid, HW, width at half-amplitude, MK-801, (5*S*,10*R*)-(+)-5-methyl-10,11-dihydro-5*H*-dibenzo[*a*,*d*]cyclohepten-5,10-imine maleate, PB, phosphate buffer, PPR, paired-pulse ratio, RT, rise time, EPSP, IPSP, interneuron, NMDA, synapse, cortex

## Abstract

To investigate the involvement of N-Methyl-D-aspartate (NMDA) receptors in local neocortical synaptic transmission, dual whole-cell recordings – combined with biocytin labelling – were obtained from bitufted adapting, multipolar adapting or multipolar non-adapting interneurons and pyramidal cells in layers II–V of rat (postnatal days 17–22) sensorimotor cortex. The voltage dependency of the amplitude of Excitatory postsynaptic potentials (EPSPs) received by the three types of interneuron appeared to coincide with the interneuron subclass; upon depolarisation, EPSPs received by multipolar non-adapting interneurons either decreased in amplitude or appeared insensitive, multipolar adapting interneuron EPSP amplitudes increased or appeared insensitive, whereas bitufted interneuron EPSP amplitudes increased or decreased. Connections were challenged with the NMDA receptor antagonist d-(−)-2-amino-5-phosphonopentanoic acid (d-AP5) (50 μM) revealing NMDA receptors to contribute to EPSPs received by all cell types, this also abolished the non-conventional voltage dependency. Reciprocal connections were frequent between pyramidal cells and multipolar interneurons, and inhibitory postsynaptic potentials (IPSPs) elicited in pyramidal cells by both multipolar adapting and multipolar non-adapting interneurons were sensitive to a significant reduction in amplitude by d-AP5. The involvement of presynaptic NMDA receptors was indicated by coefficient of variation analysis and an increase in the failures of transmission. Furthermore, by loading MK-801 into the pre- or postsynaptic neurons, we observed that a reduction in inhibition requires presynaptic and not postsynaptic NMDA receptors. These results suggest that NMDA receptors possess pre- and postsynaptic roles at selective neocortical synapses that are probably important in governing spike-timing and information flow.

## Highlights

► We investigated the involvement of NMDA receptors in synaptic transmission. ► The voltage dependency of EPSPs was target interneuron dependent. ► Postsynaptic NMDA receptors contributed to EPSPs elicited in interneurons studied. ► Blocking NMDA receptors abolished non-conventional voltage dependency. ► Presynaptic NMDA receptors modulate inhibition in pyramidal cells.

## Introduction

Local excitatory input to interneurons of the neocortex is mainly provided by pyramidal cells. Pyramidal cells are the most abundant neurons of the neocortex comprising approximately 80–90% of the entire population, while GABAergic interneurons make up 10–20% of neurons (Ascoli et al., 2008). The glutamatergic pyramidal cells are a relatively homogenous group when compared to interneurons, which are a highly diverse population and, as such, are classified based upon a wide range of criteria (Markram et al., 2004; Thomson and Lamy, 2007; Ascoli et al., 2008). These criteria encompass molecular, anatomical and electrophysiological properties as well as the role of interneurons in network activity.

Excitatory input to different classes of interneuron is synapse specific and a single neocortical pyramidal cell can provide two different forms of short-term plasticity: depression and facilitation, depending on the class of postsynaptic interneuron (Reyes et al., 1998; Rozov et al., 2001). Bitufted interneurons, at which tufts of dendrites extend from the apical and basal poles, primarily receive EPSPs that facilitate, while EPSPs elicited in multipolar interneurons – dendrites radiate from around the soma – depress. In addition to differences in synaptic dynamics at specific interneuron classes, the EPSPs they receive also vary in magnitude and time course (Ali et al., 2007). Influencing these properties are presynaptic factors including the probability of vesicle release, quantal content and vesicle composition, and postsynaptic factors such as the type, density and location of ionotropic glutamate receptors (Conti and Weinberg, 1999).

Ionotropic glutamate receptors, namely Alpha-Amino-3-Hydroxy-5-Methyl-4-Isoxazole Propionic (AMPA), kainate and NMDA are widely distributed throughout the CNS and mediate fast excitatory synaptic transmission (Dingledine et al., 1999; Traynelis et al., 2010). NMDA receptors have become attractive therapeutic targets due to their involvement in neurological disorders (Kalia et al., 2008; Benarroch, 2011). Functional NMDA receptors are heterotetramers composed of two GluN1 subunits and at least one type of GluN2 and/or GluN3 subunit, which form non-selective cation channels permeable to Na^+^, K^+^ and Ca^2+^. Under normal physiological conditions, Mg^2+^ blocks the channel pore in a voltage-dependent manner. When the cell is depolarised, Mg^2+^ blockade is relieved, which may play a role in shaping EPSPs and bestowing a non-conventional voltage dependence upon them. In addition to their postsynaptic role, anatomical evidence using immuno-electron microscopy and electrophysiological studies suggest a presynaptic location of NMDA receptors within the entorhinal cortex (Berretta and Jones, 1996) and the neocortex, where they may modulate glutamatergic and GABAergic transmission (Aoki et al., 1994; DeBiasi et al., 1996; Sjöström et al., 2003; Bender et al., 2006; Corlew et al., 2007; Brasier and Feldman, 2008; Mathew and Hablitz, 2011) and are considered to be important for spike-timing plasticity (Rodríguez-Moreno and Paulsen, 2008; Larsen et al., 2011).

In the present study, we performed dual whole-cell recordings, combined with biocytin labelling to obtain synaptic connections between pyramidal cells and interneurons in order to investigate: (i) the properties of EPSPs at excitatory synapses formed with three subclasses of interneurons and (ii) the contribution of NMDA receptors to excitatory and inhibitory neurotransmission. Our results indicate that NMDA receptors contribute to EPSPs received by bitufted adapting, multipolar adapting and multipolar non-adapting interneurons and play a presynaptic role in enhancing inhibition between multipolar interneurons and pyramidal cells.

## Experimental procedures

### Slice preparation

All procedures were performed in accordance with UK Home Office regulations under the Animals (Scientific Procedures) Act 1986. Male Wistar rats (Harlan, UK), at postnatal days 17–22, were anaesthetised via an intraperitoneal injection of sodium pentobarbitone (60 mg kg^−1^; Euthatal, Merial, UK). Rats were perfused transcardially with 50–100-ml of ice-cold sucrose containing Artificial cerebrospinal fluid (ACSF) consisting of (in mM): 248 sucrose, 3.3 KCl, 1.4 NaH_2_PO_4_, 2.5 CaCl_2_, 1.2 MgCl_2_, 15 glucose and 25.5 NaHCO_3_ bubbled with 95% O_2_ and 5% CO_2_. Following decapitation and removal of the brain, 300-μm coronal sections of cortex were cut using an automated vibratome (Leica, Germany) in an ice-cold standard ACSF solution containing (in mM): 121 NaCl, 2.5 KCl, 1.3 NaH_2_PO_4_, 2 CaCl_2_, 1 MgCl_2_, 20 glucose and 26 NaHCO_3_ equilibrated with 95% O_2_ and 5% CO_2_. Slices were incubated in ACSF for 1 h prior to recording at room temperature, for which slices were placed in a submerged chamber perfused with ACSF at a rate of 1–2-ml min^−1^.

### Dual whole-cell recordings

Dual whole-cell somatic recordings were obtained from layer II–V interneurons and pyramidal cells of rat sensorimotor cortex. Recordings were carried out in the current clamp mode of operation (SEC 05LX amplifiers, npi electronics, Germany), low pass filtered at 2 kHz and digitised at 5 kHz using a CED 1401 interface (Cambridge Electronic Design, UK). The software Signal (Cambridge Electronic Design, UK) was used to acquire recordings and deliver stimuli. Electrodes with resistances of 8–12 MΩ were pulled from borosilicate glass (Harvard Instruments, UK) and filled with a solution containing (in mM): 134 K^+^ gluconate, 10 HEPES, 10 phosphocreatine, 2 Na_2_ATP, 0.2 Na_2_GTP, and 0.2% (w/v) biocytin.

Slices were viewed using near infrared differential interference contrast video microscopy, allowing for the selection of cells based upon the shape of their soma and dendritic projections. Neurons were further characterised by their electrophysiological properties obtained from a series of 500-ms depolarising and hyperpolarising current pulses ranging from −0.25 nA to +0.20 nA, and from their neuroanatomy revealed through biocytin labelling (see below for histological processing and reconstruction of recorded cells). The existence of a connection between an interneuron and pyramidal cell was tested by inducing an action potential in the presynaptic cell and observing the response in the postsynaptic cell. A single action potential was elicited by injecting a short square pulse of positive current (+0.05 nA, 5–10 ms), repeated at 0.33 Hz. The presence of a connection was tested in both directions to determine if it was of a reciprocal nature. To elicit a paired-pulse effect, the initial current injection was followed by an identical stimulus 50 ms later, and to observe brief train effects, a square pulse of +0.2 nA for 200 ms was used. When recording EPSPs, interneurons were held at a membrane potential between −54 and −56 mV and −68 and −71 mV, and for IPSPs, between −50 and −55 mV. IPSPs recorded had a reversal potential around −70 mV, indicating they were GABA_A_ receptor mediated. IPSPs elicited by multipolar cells were usually more sensitive to somatic voltage changes.

Input and series resistance were monitored continuously by applying brief hyperpolarizing pulses of −2.5 mV for 10 ms at the start of each frame and 100 ms before synaptic events were triggered. The series resistance which fell in the range of 10–30 MΩ and the input resistance were between 220 and 300 MΩ.

The competitive NMDA receptor antagonist d-(−)-2-amino-5-phosphonopentanoic acid (d-AP5) (Tocris, UK) was dissolved in distilled water, stored as stock at −20 °C, and for experiments, delivered in the ACSF at a concentration of 50 μM. The non-competitive NMDA receptor antagonist (MK-801) (Tocris, UK) was dissolved in distilled water (stock solution, 25 mM, stored at −20 °C) and added to the intracellular solution (1 mM).

### Data analysis

The selection criteria for including electrophysiological recordings were based on stable membrane potential, series and input resistances. If series resistance (range, 10–30 MΩ) and input resistances (range, 220 and 300 MΩ) changed by more than 15% of the original values, recordings were excluded from analysis. Presynaptic membrane potential was, −70 ± 2.5 mV and postsynaptic potentials were in the range of −50 and −80 mV (EPSPs were recorded at subsets of membrane potentials). The postsynaptic membrane potential was carefully monitored throughout the experiments and was not allowed to deviate ±0.5 mV from original value. Data sets included in this manuscript were recordings of duration of 30 and 90 min obtained together with identification of recorded cell pairs.

However, for electrophysiological properties shown in Table 2, data were pooled from recorded cells which were not all included in the detailed morphological analysis shown in Table 1, either because of faint biocytin labelling or recording being less that 30 min, although the intrinsic membrane properties were correlated with cells identified as bitufted or multipolar cells.

Data were analysed off-line using the software Signal and data sets in which the first EPSP/IPSP shape and amplitude and the postsynaptic membrane potential were stable, were selected. Single sweeps were checked individually to ensure that every presynaptic action potential was recognised by the software and that the trigger points used for subsequent analysis were accurately aligned with the fast component of the rising phase of each action potential. Sweeps that included artefacts or large spontaneous events were excluded from the averaged records. The amplitude of EPSPs and IPSPs was measured from the baseline to the peak of single sweep events, while the postsynaptic 10–90% rise time (RT) and width at half-amplitude (HW) were measured from averaged data. Averages were created from 35 to 150 sweeps. Averages obtained after bath application of D-AP5 or internal solution containing MK-801 were from data sets where amplitudes of the synaptic events reached a plateau. The paired-pulse ratio (PPR) was calculated by dividing the second average EPSP amplitude by the first average EPSP amplitude.

The amplitudes were measured for each individual EPSP obtained at membrane potentials held at the most depolarised (−55 ± 1 mV) and hyperpolarised (69 ± 1 mV) potentials, and these were then averaged to determine a value for each connection at those membrane potentials. While some connections displayed an increase in peak amplitude with increasing depolarisation, others displayed a decrease, most likely due to the differential involvement of AMPA and NMDA receptors. To ask whether the ratio of AMPA to NMDA components remained constant after different experimental conditions, we determined the hyperpolarised–depolarised EPSP ratio for each individual connection. We define this normalisation of the amplitudes as the voltage ratio. The failure of a presynaptic action potential to elicit an EPSP or IPSP was measured from 100 sweeps and is shown as the failure rate (%). Apparent failures of synaptic transmission were assessed by eye and counted manually. Selection and averaging of these apparent failures resulted in an inadequate signal-to-noise ratio and no measurable postsynaptic response obtained.

Data are presented as the mean ± standard deviation (SD). An unpaired *t* test was used to compare two groups of data. For comparisons between more than two groups of data, a one-way analysis of variance (ANOVA) was used and a Bonferroni correction imposed for multiple comparisons. A paired *t* test was used to compare groups of data before and after application of d-AP5. For statistical tests, significance was accepted if *p *< 0.05.

### Histological processing and reconstruction of recorded cells

Immediately following the termination of recording, slices were immersed in fixative containing 4.0% paraformaldehyde, 2.5% gluteraldehyde and 0.2% picric acid in 0.1 M phosphate buffer (PB), and stored overnight at 4 °C. Slices were embedded in 12% gelatin, fixed, and cut into thinner, 60 μM sections. Prior to freeze thawing just above the surface of liquid nitrogen (3 × 30 s), sections were submerged for cryoprotection in a series of 0.1 M PB solutions containing: 10% sucrose for 2 × 10 min, 20% sucrose and 6% glycerol for 2 × 20 min, followed by 30% sucrose and 12% glycerol for 2 × 30 min. Sections were incubated in 1% hydrogen peroxide for 30 min and immersed overnight in a preformed avidin and biotinylated horseradish peroxidase solution (Vectastain Elite ABC Kit; Vectorlabs, US). Cells were visualised by incubating sections in 3,3′-diaminobenzidine and nickel chloride for 15 min, followed by the addition of 1% hydrogen peroxide. Osmium tetroxide (1%) was used to intensify staining prior to dehydration of the sections in an ascending series of ethanol solutions (50% for 2 × 10 min, 70% for 2 × 20 min, 95% for 2 × 20 min and 100% for 30 min) and immersion in propylene oxide. Sections were embedded in epoxy resin (Durcupan; Fluka, Switzerland).

Biocytin-labelled cells were reconstructed using a microscope with an attached drawing tube (Olympus, UK), at a magnification of 1000×, utilising a 100× oil immersion objective lens (Olympus, UK). Three-dimensional reconstructions of the soma and dendrites of neurons were produced using Neurolucida (MBF Bioscience, US). To compare the relative length of axon between groups of interneurons, the length of axon was measured within a 60-μm section containing the soma, by means of a 100-μm × 100-μm square centred to the cell body. Neuroanatomical measurements were obtained from slides and have not been corrected for shrinkage which may occur during processing of the slices.

## Results

### Neuroanatomical and electrophysiological properties of three types of interneurons

**Fig. 1:**
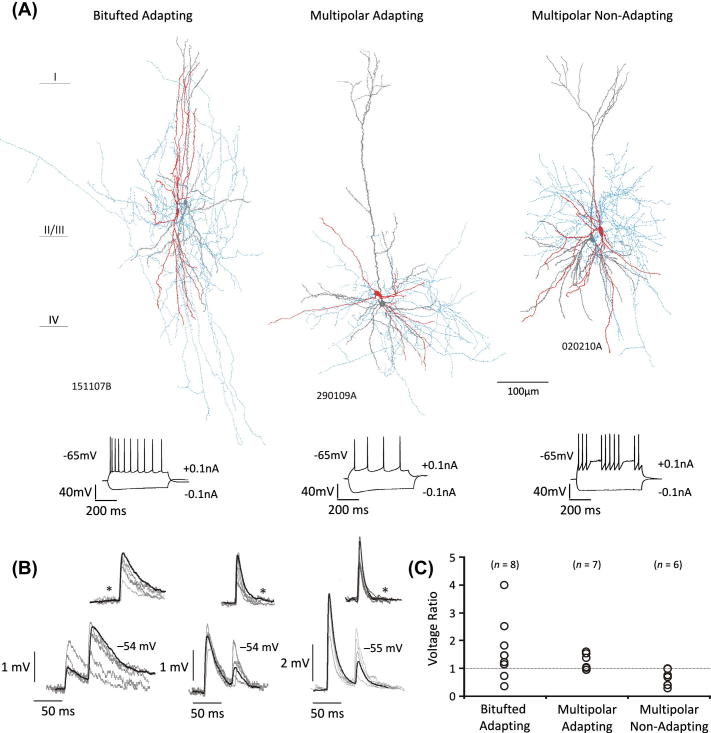
EPSPs received by bitufted adapting, multipolar adapting and multipolar non-adapting interneurons, and their voltage dependency. (A) Biocytin-labelled bitufted adapting, multipolar adapting and multipolar non-adapting interneurons with their respective firing pattern and presynaptic pyramidal cell. Interneuron soma and dendrites in red, axon in blue and pyramidal cell dendrites in grey. (B) EPSPs received by the three interneurons shown in A. Examples of single data are superimposed (grey traces) with average trace (black trace) for each class of synapse studied. Inserts show examples of apparent synaptic failures observed (asterisk denotes a failure), similarly five single sweep traces (grey) are superimposed with the averaged trace (black). (C) The voltage ratio (change in amplitude dependent on the holding potential of the cell, see “Data analysis”) of EPSPs received by bitufted adapting, multipolar adapting and multipolar non-adapting interneurons. Each circle represents one pair.

Interneurons were classified depending firstly on their dendritic anatomy into traditional bitufted (excluding bipolar and Martinotti cells) and multipolar groups, and secondly, by their electrophysiological properties to form bitufted adapting, multipolar adapting and multipolar non-adapting groups (Ali and Thomson, 2008). Examples of each cell type are shown in Fig. 1A.

The neuroanatomical properties of bitufted and multipolar interneurons are summarised in Table 1. Bitufted cells possessed somata that were round, oval or fusiform, and longer in the vertical orientation than those of the multipolar interneurons (unpaired *t* test, *p* < 0.01), which were a variety of shapes. Tufts of branches extended from each pole of the soma of bitufted interneurons, whereas the dendrites of multipolar interneurons frequently radiated from around the soma. On average, the bitufted interneurons possessed a smaller number of primary dendrites, a larger mean total dendritic length and a greater number of mean branch points per dendrite compared with the multipolar group (unpaired *t* test, *p *< 0.01). Although the relative length of axon (see “Experimental procedures”) was greater in the multipolar group (unpaired *t* test, *p *< 0.01), it was the bitufted group which possessed the greatest span of axon in the vertical direction (unpaired *t* test, *p *< 0.01), while in the horizontal direction the span was similar between the two groups.

The electrophysiological properties of the three groups of interneuron are summarised in Table 2. There was little difference in these between the bitufted adapting and multipolar adapting interneuron groups; however, multipolar non-adapting interneurons possessed a lower input resistance, shorter membrane time constant and less frequency adaptation (Bonferroni, *p* < 0.05), as calculated from a train of action potentials in response to a 500-ms depolarising current step, by dividing the final interspike interval by the first. A narrow action potential HW in addition to a large after-hyperpolarisation (AHP) amplitude was also a characteristic of the multipolar non-adapting cells (Bonferroni, *p *< 0.05).

### Properties and voltage dependency of EPSPs received by bitufted adapting, multipolar adapting and multipolar non-adapting interneurons

The properties of EPSPs received by the three types of interneuron are shown in Table 3. Multipolar non-adapting interneurons received EPSPs with the fastest 10–90% RT (Bonferroni, *p *< 0.05) and HW, while those received by the bitufted interneurons were the slowest and smallest in amplitude. The mean failure rate was highest in the bitufted adapting group (Bonferroni, *p *< 0.05). With a presynaptic interspike interval of 50 ms, all multipolar interneurons included in this study received EPSPs displaying paired-pulse depression (PPD), with the strongest depression observed at the pyramidal cell to multipolar non-adapting connections. In contrast, EPSPs received by bitufted adapting interneurons demonstrated paired-pulse facilitation (PPF).

EPSPs elicited by pyramidal cells displayed three types of response in amplitude as a reaction to changes in the holding potential of the postsynaptic cell – upon depolarisation, mean amplitudes increased (non-conventional voltage relation), decreased (conventional voltage relation) or appeared insensitive to the change in membrane potential. Individual connections were deemed insensitive by the lack of a significant change in the EPSP amplitude (unpaired *t* test, *p* ⩾ 0.05). The voltage dependence appeared to coincide with the type of postsynaptic interneuron, with multipolar adapting interneurons displaying a voltage ratio (see “Data analysis”) >1, while multipolar non-adapting interneurons possessed a voltage ratio <1 (Fig 1C). Both groups also contained pairs with average EPSPs that were insensitive. Average EPSPs received by bitufted adapting interneurons either increased or decreased upon depolarisation.

### NMDA receptors contribute to EPSPs elicited in bitufted adapting, multipolar adapting and multipolar non-adapting interneurons

**Fig. 2:**
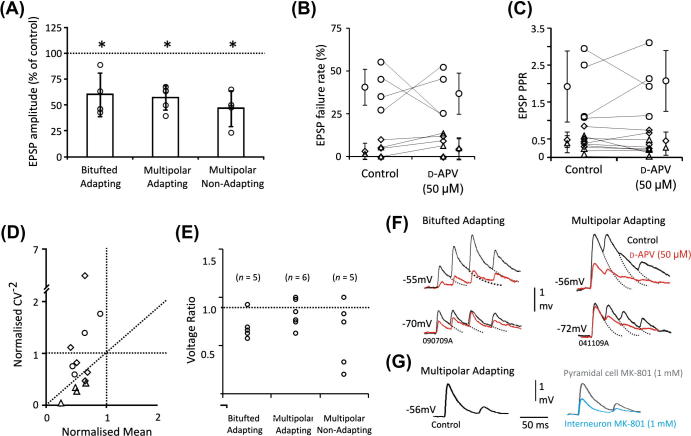
NMDA receptors contribute to EPSPs received by bitufted adapting, multipolar adapting and multipolar non-adapting interneurons. (A) Average EPSPs received by all three interneuron groups were significantly reduced by d-AP5 (50 μM, paired *t* test, *p* < 0.05). Each circle represents a pair, while the bars represent the group average. Error bars denote SD. (B) The change in the failure rate with d-AP5 shown for individual pairs and (C) the paired-pulse ratio (PPR) during control and with d-AP5 shown for individual pairs. The group average is shown for each interneuron group with bars representing SD. (D) Normalised (d-AP5/control) plot of the CV^−2^ against the normalised mean for the three cell types. A greater change in the mean than CV^−2^ indicates a postsynaptic mechanism. (E) The voltage ratio with d-AP5 for the three types of connection (individual circles represent a pair). (F) EPSPs elicited in bitufted and multipolar adapting interneurons at depolarised and hyperpolarised potentials during control and with d-AP5. The voltage relation becomes conventional when NMDA receptors are blocked with d-AP5. In B–D, circles represent bitufted pairs, diamonds represent multipolar adapting pairs and triangles represent multipolar non-adapting pairs. (G) EPSPs elicited in a mulitipolar adapting in control conditions, after repatching the presynaptic pyramidal cells with the NMDA receptor antagonist, MK-801 (1 mM, grey traces), and then subsequently repatching the postsynaptic interneurons with MK-801 (blue traces). (H) The average EPSP amplitudes (% of control) of each individual pair with MK-801 in the pipette of the pyramidal cells and interneurons.

On addition of the competitive NMDA receptor antagonist d-AP5 (50 μM), the mean EPSP amplitude obtained from interneurons depolarised to a value between −54 mV to −56 mV was decreased to 60.31 ± 21.06% of control for the bitufted adapting group (*n *= 4), 57.14 ± 11.18% (*n *= 5) for the multipolar adapting group and 46.69 ± 17.25% for the multipolar non-adapting group (*n *= 4). This reduction was statistically significant in all three groups (paired *t* test, *p *< 0.05; Fig. 2A), implying that NMDA receptors contribute to EPSPs received by all three groups of interneuron. A statistically significant difference in the reduction in amplitude between the three groups was not found (Bonferroni, *p* ⩾ 0.05). The decrease in the amplitudes of the EPSPs in all three groups with d-AP5 was without coincident changes in the holding membrane potential, series resistance, or input resistance.

The EPSP failure rates and PPR did not change significantly in all three groups with d-AP5 (paired *t* test, *p* ⩾ 0.05), although see data from individual connections in Fig. 2B and C. The failure rates changed from 40.50 ± 12.15% to 36.75 ± 13.85%, from 3.00 ± 4.47% to 4.20 ± 5.85% and from 1.25 ± 2.50% to 4.75 ± 6.18% of control for bitufted adapting, multipolar adapting and multipolar non-adapting cells, respectively. On average, the PPR in d-AP5 was 108 ± 82%, 94 ± 55% and 66 ± 75% of control EPSPs for bitufted adapting, multipolar adapting and multipolar, non-adapting cells, respectively.

A pre- or postsynaptic site of action by d-AP5 may be indicated, when assuming a simple binomial model of synaptic release, by changes in the mean amplitude (*M*) relative to changes in the inverse square of the coefficient of variation (CV^−2^). Where *M* = *npq* and CV^−2^ = *np*/(1 − *p*), *n* being the number of release sites, *p* the probability of release and *q* the quantal amplitude. A change in *M* with no change in CV^−2^ indicates a change in *q* and a postsynaptic mechanism. A greater change in CV^−2^ than in *M* suggests a change in *p*, whereas an equivalent proportional change in both CV^−2^ and *M* indicates a change in *n*. Fig. 2D shows a plot of the normalised CV^−2^ (d-AP5/control) against the normalised *M* (d-AP5/control). A greater change in the CV^−2^ than in *M* is seen for all of the multipolar non-adapting pairs suggesting a change in the probability of release, while a mixed response is seen in the bitufted adapting and multipolar adapting groups.

Upon depolarisation with d-AP5, mean amplitudes decreased and individual connections in all three groups illustrated a conventional voltage relation (Fig. 2E and F), suggesting that NMDA receptors contributed to the unusual insensitive and non-conventional voltage relationships.

To confirm that the changes in the peak amplitudes of the EPSPs were due to activation of postsynaptic NMDA receptors and not presynaptic NMDA receptors at the excitatory synapses studied, MK-801 (1 mM), a use-dependent non-competitive NMDA receptor antagonist was loaded into the presynaptic neuron followed by the postsynaptic neuron during paired recordings. Loading of MK-801 into the presynaptic pyramidal cells had no affect and the amplitudes remained at 100 ± 2% of the control amplitude. However, when loading MK-801 into the postsynaptic multipolar (adapting or non-adapting) and bitufted interneurons, the amplitudes of the EPSPs reduced by 51 ± 3% of the control amplitude (paired *t* test, *p *< 0.05; *n* = 3, pooled data sets). Fig. 2G illustrates an example of a pyramidal and multipolar adapting connection with MK-801 (see Fig. 2H for individual pairs with MK-801).

### Presynaptic NMDA receptors facilitate inhibition between multipolar interneurons and pyramidal cells

**Fig. 3:**
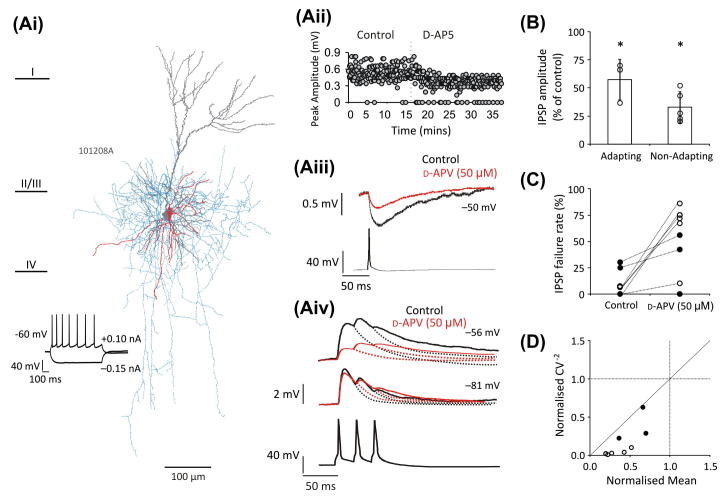
NMDA receptors enhance inhibition between multipolar interneurons and pyramidal cells. (Ai) Example of a biocytin-labelled multipolar adapting interneuron and pyramidal cell that were reciprocally connected. Interneuron soma and dendrites in red, axon in blue and pyramidal cell dendrites in grey. (Aii) Plot of an experiment showing peak amplitude of IPSPs in control and after bath application of D-AP5 (50 μM) against time, and (Aiii) the average IPSP. (Aiv) EPSPs were reduced in the presence of d-AP5. (B) The effect of d-AP5 on the amplitude of multipolar adapting and multipolar non-adapting interneuron containing pairs. Each circle represents a pair, while the bars represent the group average. Error bars denote SD. Both groups were significantly reduced from control (paired *t* test, *p *< 0.05). (C) d-AP5 (50 μM) increased the IPSP failure rate in seven out of the eight pairs. Multipolar adapting pairs marked by a black circle, multipolar non-adapting interneurons by a hollow circle. (D) Plot of the normalised (d-AP5/control) CV^−2^ against the normalised (d-AP5/control) mean. A greater change in the CV^−2^ compared with that of the mean indicates a presynaptic mechanism of action. Each point represents a single pair. As before, multipolar adapting pairs represented by a black circle, multipolar non-adapting pairs by a hollow circle.

To investigate as to whether NMDA receptors were contributing to inhibition, unitary IPSPs were recorded from reciprocal connections between multipolar interneurons and pyramidal cells, and challenged with d-AP5 (50 μM). Fig. 3Ai shows an example of a reciprocal connection between a multipolar adapting interneuron and pyramidal cell, at which, the average IPSP amplitude is reduced on addition of d-AP5. Fig. 3Aii illustrates the plot of the peak IPSP amplitudes against time in control and bath application of d-AP5, averages shown in Fig. 3Aii. A reduction in the average IPSP amplitude was observed at all pairs tested, with a significant reduction in the group means to 57.60 ± 18.23% and 32.84 ± 13.88% of control, at multipolar adapting (*n *= 3) and multipolar non-adapting (*n *= 5) interneuron containing pairs, respectively (Fig. 3B, paired *t* test, *p *< 0.01). An increase in the failures of transmission accompanying the reduction in IPSP amplitude was observed at two out of three multipolar adapting pairs and at all multipolar non-adapting pairs (Fig. 3C), and was statistically significant in the multipolar non-adapting group (paired *t* test, *p *< 0.01, *n *= 5), signifying a presynaptic site of action. A presynaptic site of action for the decrease of the mean IPSP amplitudes by d-AP5 at both the multipolar adapting and multipolar non-adapting interneuron containing pairs is also indicated by a greater change in the CV^−2^ in relation to that of *M* (Fig. 3D), suggesting that NMDA receptors enhance inhibition between multipolar interneurons and pyramidal cells by increasing the probability of neurotransmitter release.

**Fig. 4:**
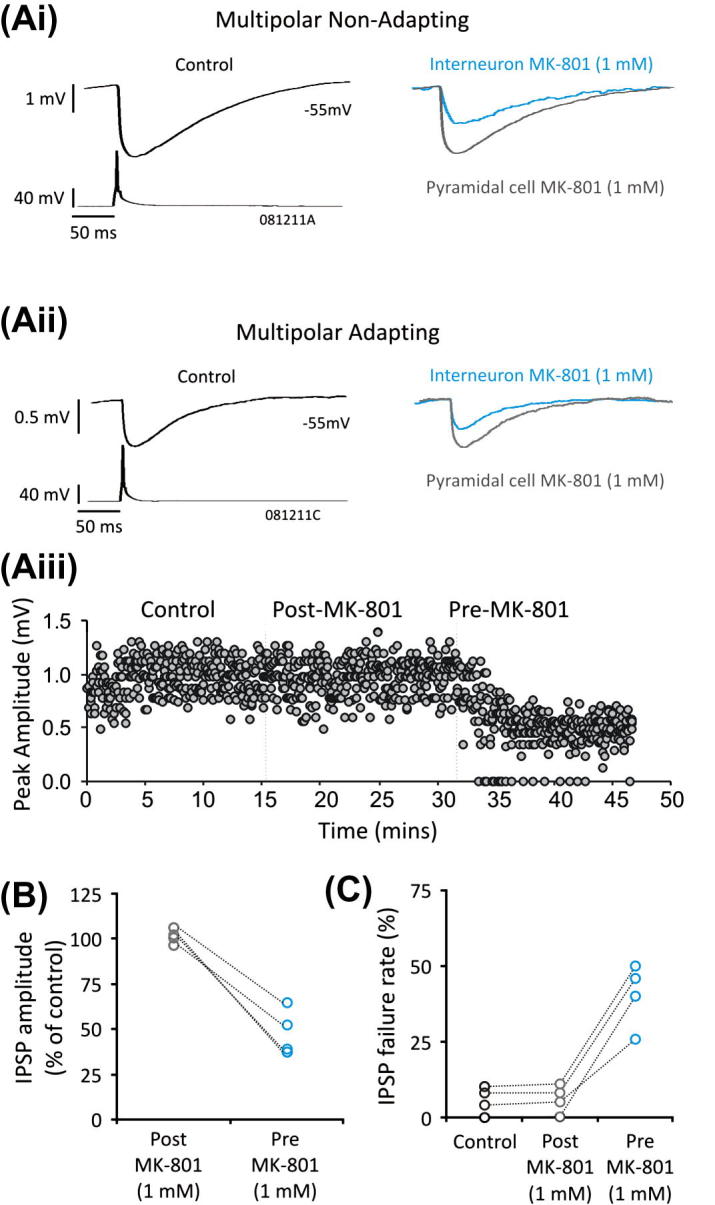
Presynaptic NMDA receptors enhance inhibition between multipolar interneurons and pyramidal cells. (Ai and Aii) IPSPs elicited by a mulitipolar non-adapting and adapting interneuron in control conditions, after repatching the postsynaptic pyramidal cells with the NMDA receptor antagonist, MK-801 (1 mM, grey traces), and then subsequently repatching the presynaptic interneurons with MK-801 (blue traces). (Aiii) Plot of an example experiment shown in (Aii), during control, repatching the pyramidal cell with Mk-801 and subsequent repatching the presynaptic interneuron with MK-801 (vertical lines indicate the onset of MK-801 application). (B) The average IPSP amplitudes (% of control) and failure rates of each individual pair with MK-801 in the pipette of the pyramidal cells and interneurons. Pre and post refer to the presynaptic interneuron and postsynaptic pyramidal cell, respectively. (For interpretation of the references to colour in this figure legend, the reader is referred to the web version of this article.)

To investigate further as to whether presynaptic NMDA receptors were involved in reducing IPSP amplitudes elicited by multipolar cells, MK-801 (1 mM), was loaded into the postsynaptic neuron followed by the presynaptic neuron during paired recordings. The mean IPSP amplitudes after postsynaptic and presynaptic loading of MK-801 were 100.72 ± 3.93% and 47.91 ± 12.95% of control, respectively (Fig. 4B, *n *= 4, data pooled for both subclasses of multipolar IPSPs). Examples of two recordings (one non-adapting and adapting containing pair) are shown in Fig. 4Ai and Aii. The presynaptic loading of MK-801 caused an effect between 3 and 5 min, and was required to significantly decrease the IPSPs (paired *t* test, *p *< 0.05, *n *= 4). This decrease was accompanied by an increase in the failure rate to 40.50 ± 10.50% (control failure rate 5.50 ± 4.43%, paired *t* test, *p *< 0.05, *n *= 4), illustrated in Fig. 4C (see also graph Aiii). The failure rate after postsynaptic loading of MK-801 was 6.00 ± 4.69% and comparable to control condition (paired *t* test, *p* ⩾ 0.05).

## Discussion

To study the properties of EPSPs elicited by pyramidal cells and received by different classes of interneuron, and to investigate the role of NMDA receptors in excitatory and inhibitory neurotransmission, dual whole-cell recordings from interneurons and pyramidal cells were combined with the biocytin labelling of neurons. Our results indicate that EPSPs demonstrate a voltage dependency that appears to coincide with the class of interneuron, which would allow for excitation to be differentially amplified or dampened in a postsynaptic target cell-type specific manner. NMDA receptors were shown to contribute to EPSPs received by all three groups of interneuron, the voltage dependency and analysis indicated that NMDA receptors may contribute to EPSPs presynaptically and postsynaptically. We found that presynaptic NMDA receptors contribute to enhancing IPSPs between multipolar interneurons and pyramidal cells.

### EPSP voltage relations and the NMDA receptor

Upon depolarisation, EPSPs received by multipolar non-adapting interneurons either decreased in amplitude or there was no significant change, with amplitudes appearing insensitive. This was similar to previously published work, which reported EPSCs at connections between layer V pyramidal cells and fast spiking interneurons in rat motor cortex to be greatest in amplitude at hyperpolarised potentials (Angulo et al., 1999). Multipolar adapting interneurons received EPSPs with amplitudes that increased upon depolarisation or appeared insensitive to changes in voltage, whereas bitufted adapting interneuron EPSP amplitudes either increased or decreased upon depolarisation. This difference in the voltage dependency of EPSPs allows excitation to be amplified or dampened depending on the activity of the interneuron and confers a functional difference between networks of different types of interneuron.

It has been previously reported that EPSPs received by neocortical layer V pyramidal cells are increased in amplitude by blocking postsynaptic small conductance Ca^2+^-activated K^+^ channels, for which the source of Ca^2+^ for activation may be from NMDA receptors, VGCCs or IP_3_ sensitive calcium stores (Faber, 2010). EPSPs are also enhanced by Na^+^ currents when pyramidal cells are held at depolarised subthreshold potentials (González-Burgos and Barrionuevo, 2001). In our study d-AP5 reduced the mean EPSP amplitude in all cell groups, suggesting that the three types of interneuron received EPSPs mediated by NMDA receptors. Our data suggest that NMDA receptors bestow the voltage dependency upon EPSPs, as blocking NMDA receptors generated a more conventional voltage dependency at all connections studied, therefore we can exclude the possibility that Na^+^ currents cause the unusual voltage dependency. The increase in EPSP amplitude upon depolarisation is due to a greater proportion of NMDA receptors activated at depolarised potentials following relief of the Mg^2+^ block, thus giving rise to larger EPSPs. This may be also influenced by the subunit composition that effect permeability, sensitivity to block by Mg^2+^, receptor kinetics and sensitivity to ligands of the receptor. Additionally, the NMDA receptor location at the synapse and a synapse-specific expression of functional NMDA receptors could explain the changes we observe. In the study by Monyer et al. (1994), a cell type specific expression of the NR2 subunits was observed within the hippocampus, where pyramidal cells expressed NR2A and NR2B mRNA, while NR2C and NR2D mRNA expression was found in interneurons. In rat neocortex, expression of NR2D subunit mRNA has been found in SOM, PV and glutamic acid decarboxylase (GAD) 67 expressing interneurons (Standaert et al., 1996). Since receptors composed of different subunits display different properties, differential expression amongst subtypes of interneuron may bestow NMDA receptor properties in a cell type-specific manner. Furthermore the NR2B-containing NMDA receptors have been shown to enhance AMPA-mediated excitation between excitatory cells in somatosensory cortex, (Brasier and Feldman, 2008), while the NR3A-subunit is thought to enable presynaptic NMDA receptors to promote spontaneous and elicited glutamate release at excitatory synapses during development (Larsen et al., 2011).

### Presynaptic NMDA receptors and the modulation of neocortical inhibition

IPSPs recorded from pyramidal cells reciprocally connected with multipolar adapting and multipolar non-adapting interneurons, were significantly reduced in amplitude on addition of d-AP5 (50 μM). Experiments performed with MK-801 and in addition, CV^−2^ analysis and an increase in the apparent failures of transmission at the majority of connections indicated that under control conditions, presynaptic NMDA receptors facilitate GABAergic transmission between these two types of interneuron and pyramidal cell. It could be that d-AP5 is acting on presynaptic NMDA receptors located on axon terminals leading to a reduction in Ca^2+^ influx and depolarisation of the presynaptic terminal, consequently lowering the probability of vesicle release either directly or indirectly (see Corlew et al., 2008). That presynaptic NMDA receptors are present and functional at GABAergic synapses within the neocortex is in agreement with a presynaptic localisation of NMDA receptor subunits at symmetrical, GABAergic synapses (DeBiasi et al., 1996). Previously, presynaptic NMDA receptors have been implicated in modulating GABA release onto pyramidal cells within layers II/III of rat frontal cortex (Mathew and Hablitz, 2011). Mathew and Hablitz (2011) propose that ambient glutamate levels are sufficient to activate presynaptic NMDA receptors, thus producing a tonic facilitation of inhibitory synaptic transmission. In addition, it was reported that application of NMDA enhanced the frequency of mIPSCs suggesting that presynaptic NMDA receptors act as a sensor for glutamate levels and adjust inhibition accordingly. Alternatively, a retrograde messenger could be involved and endocannabinoids, being a prime candidate as they are capable of modulating synaptic transmission presynaptically (Ali, 2007). It has been suggested that CB1 receptors are activated on astrocytes that can trigger the release of glutamate which would activate presynaptic NMDA receptors on synapses (Navarrete and Araque, 2008).

Furthermore, recent studies demonstrate compelling evidence of heterogeneous expression of NMDA receptors on axon terminals in both the cerebellum and visual cortex (Rossi et al., 2012; Buchanan et al., 2012). Evidence demonstrating that presynaptic NMDA receptors selectively reduce excitation elicited by single axon onto pyramidal cells, but not interneurons suggests a pathway specific expression of NMDA receptors – consistent with our study.

The presynaptic NMDA receptor-elicited increases in GABA release at target-specific local circuits have important functional implications in short- and long-term plasticity. Due to the target-specific nature of their expression, these receptors are thought to play an important role in rerouting of information flow at specific synaptic pathways during high-frequency firing – thus acting as a high-pass frequency filter (Sjöström et al., 2003; Buchanan et al., 2012). Previously, it has been reported that presynaptic NMDA receptors play an important functional role in calcium-mediated synaptic long-term plasticity and spike-timing-dependent synaptic depression that depends exclusively on presynaptic NMDA receptors and not on postsynaptic NMDA receptors (Corlew et al., 2008; Rodriguez-Moreno et al., 2010; Rossi et al., 2012). Interestingly, the NR3A subunit is thought to regulate glutamate release presynaptically and the switch to NR2B-containg presynaptic NMDA receptors is thought to be responsible for the developmental loss of spike-timing dependent long-term potentiation (Larsen et al., 2011).

In summary, our data provide evidence for selective postsynaptic and presynaptic roles for NMDA receptors at three different layer II–V neocortical inhibitory synapses. We report that the voltage dependency of EPSPs received by bitufted and multipolar interneurons is related to the interneuron subclass and the unusual voltage relation is bestowed by NMDA receptors. We also report that presynaptic NMDA receptors play an important role in tonic enhancement of inhibition at multipolar interneuron. Thus, pre- and postsynaptic NMDA receptors are thought to have different functional roles, this suggests that there is functional significance of when pre- and postsynaptic NMDA receptors engage during normal physiological activity. Our data suggest that presynaptic NMDA receptors at GABA_A_ receptor-mediated inhibitory synapses are active at rest, whereas the activation of postsynaptic NMDA receptors on excitatory synapses would be dependent on the sub-type of interneuron and the membrane potential of the interneuron activated.

These findings enhance our understanding of the multi-functional roles played by NMDA receptors in neuronal networks.

## Figures and Tables

**Table 1:** Neuroanatomical properties of bitufted and multipolar interneurons

	Bitufted (*n = *8)	Multipolar (*n = *12)
*Soma*		
Vertical diameter (μm)	35.67 ± 6.87	18.41 ± 5.86⁎
Horizontal diameter (μm)	21.0 ± 4.7	10.91 ± 1.70

*Dendrites*		
Total length (μm)	652.38 ± 132.79	257.89 ± 116.91⁎
Number of primary dendrites	3.14 ± 0.37	5.27 ± 2.13⁎
Number of branch points per dendrite	4.25 ± 0.67	2.42 ± 1.67⁎

*Axon*		
Vertical span (μm)	701.57 ± 144.67	416.50 ± 172.37⁎
Horizontal span (μm)	364.67 ± 157.74	372.0 ± 69.48
Relative length (μm)	249.50 ± 132.31	941.78 ± 630.78⁎

⁎ Significantly different from bitufted group (unpaired *t* test, *p *< 0.01).

**Table 2:** Electrophysiological properties of bitufted adapting, multipolar adapting and multipolar non-adapting interneurons. Note that the electrophysiological properties were pooled from identified interneurons which were not all included in Table 1 for detailed morphological analysis (see “Experimental procedures”).

	Bitufted adapting	Multipolar adapting	Multipolar non-adapting
Input resistance (MΩ)	338.20 ± 78.08 (*n = *7)	320.39 ± 113.31 (*n = *8)	182.04 ± 55.52⁎ (*n = *9)
Time constant (ms)	20.16 ± 6.33 (*n = *7)	20.63 ± 11.23 (*n = *8)	10.15 ± 4.40⁎ (*n = *9)
AP HW (ms)	1.69 ± 0.28 (*n = *7)	1.60 ± 0.40 (*n = *8)	0.97 ± 0.24⁎ (*n = *11)
AP AHP AMP (mV)	6.31 ± 3.58 (*n = *7)	6.35 ± 2.98 (*n = *8)	11.46 ± 3.38⁎ (*n = *11)
Frequency adaptation	3.68 ± 0.28 (*n = *5)	3.21 ± 0.81 (*n = *7)	1.34 ± 0.21⁎ (*n = *7)

AHP, after-hyperpolarisation; AMP, amplitude; AP, action potential; HW, width at half-amplitude.

⁎ Significantly different from bitufted adapting and multipolar adapting groups (Bonferroni, *p *< 0.05).

**Table 3:** Properties of EPSPs received by bitufted adapting, multipolar adapting and multipolar non-adapting interneurons.

	Bitufted adapting (*n = *8)	Multipolar adapting (*n = *7)	Multipolar non-adapting (*n = *5)
AMP (mV)	0.70 ± 0.64	1.38 ± 0.52	1.79 ± 1.82
10–90% RT (ms)	4.59 ± 1.89	3.64 ± 1.22	2.12 ± 0.28⁎
HW (ms)	26.50 ± 10.73	27.00 ± 8.39	19.00 ± 7.84
Failure rate (%)	43.25 ± 14.73†	2.14 ± 3.93	1.00 ± 2.24
PPR	1.85 ± 0.62†	0.49 ± 0.20	0.42 ± 0.18
Probability of connection	1:4	1:2	1:1

AMP, amplitude; AP, action potential; HW, width at half-amplitude; RT, rise time; PPR, paired-pulse ratio. Probability of connection ratio is the number pyramidal cells tested with a single interneuron before a synaptic connection observed.

⁎ Significantly different from bitufted adapting and multipolar adapting groups (Bonferroni, *p *< 0.05).

† Significantly different from multipolar adapting and multipolar non-adapting groups (Bonferroni, *p *< 0.05).
